# Integration and innovation: medical and health consortia improving continuing medical education in China

**DOI:** 10.3389/fpubh.2025.1633363

**Published:** 2025-10-02

**Authors:** Kang An, Jinyi Zhang, Xingyou Wang, Yi She, Shuangqing Li, Sheyu Li

**Affiliations:** ^1^General Practice Ward/International Medical Center Ward, General Practice Medical Center, National Clinical Research Center for Geriatrics, West China Hospital, Sichuan University, Chengdu, Sichuan, China; ^2^Health Promotion and Food Nutrition & Safety Key Laboratory of Sichuan Province, Chengdu, China; ^3^Chengdu Second People's Hospital, Chengdu, Sichuan, China; ^4^School of Computing, Ulster University, Belfast, United Kingdom; ^5^School of Health Policy and Management, Chinese Academy of Medical Sciences & Peking Union Medical College, Beijing, China; ^6^Jin-cheng Community Health Service Center, Chengdu, Sichuan, China; ^7^Fang-cao Community Health Service Center, Chengdu, Sichuan, China; ^8^Department of Endocrinology and Metabolism, Laboratory of Diabetes and Metabolism Research, West China Hospital, Sichuan University, Chengdu, China; ^9^MAGIC China Center, Cochrane China Center, Chinese Evidence-Based Medicine Center, West China Hospital, Sichuan University, Chengdu, China

**Keywords:** medical and health consortia, telemedicine, general practitioner, primary care, continuing medical education

## Abstract

**Background:**

Primary health care (PHC) is the cornerstone of the healthcare system in China. The medical and health consortia (medical consortia) integrate resources of continuing medical education (CME) to bridge competency gaps among healthcare providers. This narrative review aims to explore the innovative models of CME within the framework of medical consortia.

**Methods:**

Searches were conducted in both Chinese and English databases to broaden the scope of the review, including China National Knowledge Infrastructure, Wanfang Data, and PubMed. Chinese policy documents were retrieved from official websites of China’s National Health Commission. The review analyzed existing policy documents (2010–2025) and relevant literature, supplemented by an institutional application example of the West China Hospital–Fangcao Community Health Service Center Medical Consortium to explore challenges and recommendations.

**Results:**

China developed a series of policies to promote the construction of medical consortia, with a focus on resource-sharing between tertiary and PHC institutions. A literature search yielded 196 articles, including qualitative studies, quantitative studies, and reviews, of which 48 met inclusion criteria in the review. Seven policy documents were included in the analysis. The synergy between medical consortia and CME brought benefits to both healthcare providers and the health system. Key innovations included clinical scenario-oriented training, remote consultation, and flexible training modalities. However, the reviewed literature highlighted persistent challenges, including regional disparities in resources, limited financial incentives for general practitioners (GPs), and a shortage of qualified trainers. Overcoming barriers such as regional resource disparities and improving the intrinsic motivation of GPs remained critical to the implementation of CME.

**Conclusion:**

Medical consortia offer platforms for the delivery of CME, while CME supports the development of medical consortia. These innovations enhance collaboration between specialists and GPs, thereby optimizing patient referrals and follow-up care.

## Background

Primary health care (PHC) is the first element of the care continuum. In China, PHC system provides outpatient clinics, inpatient wards, and continuous preventive through general practitioner teams for community and village residents, focusing on managing common and chronic diseases (1). The goal of Healthy China 2030 aims to establish a universal basic healthcare system that provides safe, effective, convenient, and affordable PHC services to all by 2030 (2–4). However, current healthcare services in China have not yet fully achieved these objectives.

### The dilemma of China’s healthcare system

**Figure 1:**
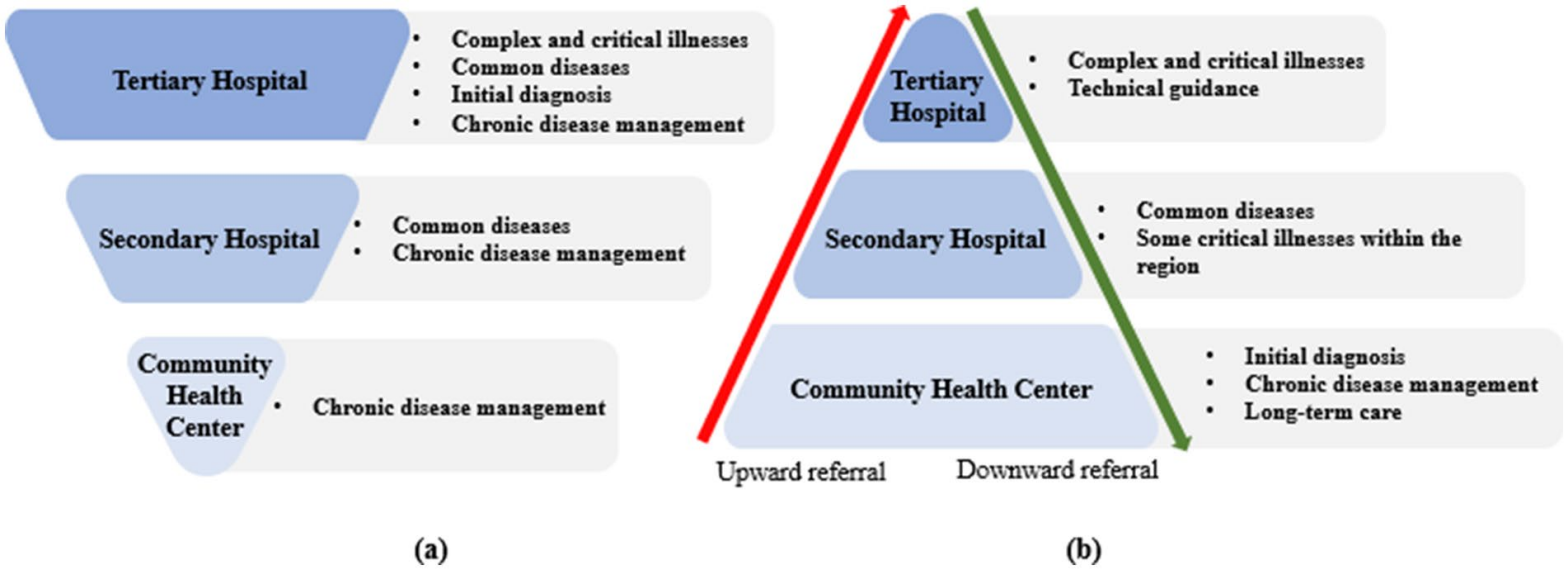
Transition from the current healthcare system to the graded diagnosis and treatment system. **a**: Current healthcare system; **b**: Graded diagnosis and treatment system.

The ongoing expansion of tertiary hospitals has led to a “siphon effect,” drawing both medical professionals and patients away from primary care. The root of the dilemma lies in the unequal distribution of medical resources (5–8). There is a small difference in medical costs and basic insurance reimbursement rates across different healthcare levels (9, 10). Patients exhibited a strong preference for seeking care at tertiary hospitals, regardless of the severity of conditions (11, 12). Tertiary hospitals are currently overburdened, expanding both outpatient and inpatient services to manage not only complex and critical illnesses but also common diseases, initial diagnoses, and chronic disease management (13, 14). This patient flow imbalance leads to a disproportionate allocation of health insurance funds toward tertiary hospitals. Meanwhile, PHC facilities remain underutilized, leading to significant inefficiencies and resource wastage (see Figure 1a) (15, 16). This imbalance is further evident in hospital admissions: in 2023, tertiary hospitals accounted for 245.001 million admissions (81.2% of the total), whereas PHC institutions managed only 45.451 million admissions (15.1%) (17). In addition, tertiary hospitals reported bed occupancy rates of 91.1%, compared to just 54.1% in PHC institutions (17).

### Structure and function of medical consortia

To eliminate the siphon effect, medical and health consortia (medical consortia) represent a key strategy to accelerate progress toward the Healthy China 2030 goals (18). Medical consortia typically consist of healthcare institutions across all three levels (tertiary, secondary, and primary care facilities). The leading institutions are usually tertiary hospitals with affiliated departments of medical education. This system allocated responsibilities appropriately across different healthcare levels (see Table 1) (19). GPs managed common and chronic diseases, while tertiary hospitals specialized in complex and critical conditions. Patients could be referred upward for advanced treatment or downward for rehabilitation and follow-up, ensuring a continuum of care (depicted in Figure 1b). The core objective of medical consortia was to direct high-quality medical resources to primary care, including continuing medical education (CME) resources (16, 20). As of the end of 2023, more than 18,000 various types of medical consortia had been established nationwide (21). While previous studies assessed the system’s management efficiency, little attention had been paid to the mechanisms of inter-institutional CME.

**Table 1:** Key tasks and implementation strategies for the medical consortia.

Task	Objective	Implementation
Downward transfer of medical resources (19)	Encourage more patients to seek care at the primary level; Reduce the burden on hospitals.	The lead tertiary hospital should allocate at least one-third of its outpatient appointments and one-quarter of inpatient beds to PHC institutions.
Upward transfer of critically Ill, complex, or contraindicated patients (19)	Ensure timely access to specialized treatment for patients.	Establish a fast-track referral pathway for transferring critical, complex, or contraindicated patients from primary care to secondary or tertiary hospitals.
Shared medical resources (19)	Optimize resource allocation; Enhance service capacity of PHC.	Shared resources include CME resources, clinical laboratories, medical imaging, electrocardiography, pathology, and sterilization services. The resources should be recognized and utilized by all institutions. Physicians should collaborate with and mentor GPs at PHC institutions.
Strengthening coordination public health (19)	Strengthen emergency response capabilities for major infectious diseases and public health crises	Facilitate public health information sharing between institutions.

CME, Continuing Medical Education.

### Overview of CME in China

CME is organized and carried out by national, provincial, and municipal health authorities, as well as hospitals, universities, and medical associations. The content covers new developments and advances in medicine, professional theories, treatment and management of common diseases, health policies and laws, new skills and practices and research capabilities (22, 23).

Prior to 2019, GPs in China were required to earn 25 CME credits, primarily for annual evaluations and professional title applications (24). The traditional approach was often characterized by seminars, hospital-based training, and self-directed learning (25). CME courses were typically conducted in large conference or seminar formats, delivering continuing education through on-the-job learning (23). Studies identified several challenges associated with CME, including fragmented learning experiences, outdated curricula, lack of learner initiative, irregular scheduling, and an excessive focus on earning credits (26). Hospital-based training was another alternative that focused on clinical skills and was typically offered at larger medical centers. However, these programs frequently failed to address the specific needs of PHC. CME activities conducted during personal time without compensation tend to reduce motivation and engagement (27). Financial barriers, such as travel costs, loss of income, and accommodation expenses, can also hindered participation in high-quality CME programs (28). The majority of GPs relied on medical textbooks for guidance in diagnosing and treating patients (29). Textbooks contained outdated or inaccurate recommendations.

Given the rapid development of medical consortia, there is an urgent need for strategies to advance CME in China. This narrative review focuses on innovative CME practices within the medical consortia and explores the interconnections.

## Methods

**Figure 2:**
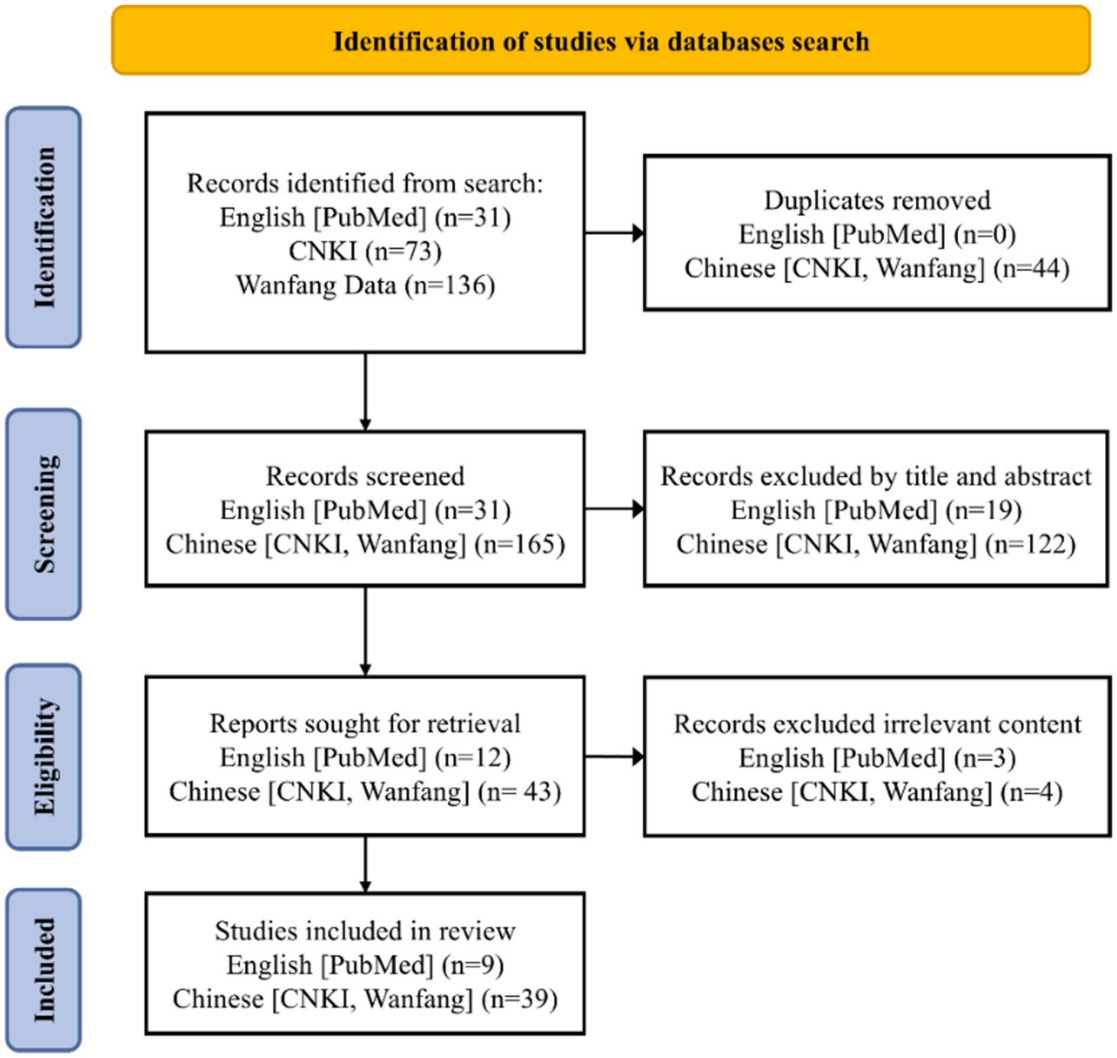
Flow diagram depicting the strategy for literature search and selection.

This narrative review analyzed existing policy documents and relevant literature, supplemented by an illustrative example of the West China Hospital–Fangcao Community Health Service Center Medical Consortium (Huaxi-Fangcao Medical Consortium), to explore the challenges and recommendations. The review employed the Joanna Briggs Institute PCC framework (population, concept, and context) to define the objectives and eligibility criteria. Searches were conducted to expand the review’s scope in both Chinese and English databases: China National Knowledge Infrastructure (CNKI), Wanfang Data, and PubMed. The primary search terms included ‘medical consortia,’ ‘medical alliance,’ ‘healthcare alliance,’ ‘medical coalition,’ ‘medical consortium,’ ‘training,’ ‘education,’ ‘learning,’ and MeSH term ‘Education.’ The search covered articles published between January 1, 2014, and April 14, 2025, because significant policy developments and pilot programs started around 2015. The inclusion criteria were: (1) publications in English or Chinese; (2) articles or reviews; (3) studies related to continuing medical education in China; (4) studies on doctor training, including general practitioners, family doctors. Exclusion criteria were: (1) studies focused on standardized training for residents, undergraduate students, postgraduates, pharmacists, or nurses; (2) articles such as conference papers, theses, commentaries, editorials, and letters. The details of the literature search were presented in Figure 2.

To identify relevant national policies and directives, we systematically searched official websites of China’s National Health Commission. These websites represent the authoritative source of healthcare policies in China. We focused on documents published between January 2010 and December 2024, corresponding to the period of major policy reforms in medical consortia and CME. We used advanced search functionalities with keywords such as medical consortia, tiered healthcare, and continuing medical education. Policy documents unrelated to CME or lacking clear directives on medical consortia implementation were excluded. The included documents were analyzed using content analysis. The institutional experiences described in the illustrative example of the Huaxi-Fangcao Medical Consortium were derived from internal practice records and routine operational documentation. It was not based on empirical research data. These records captured day-to-day operational details and administrative insights that were not typically included in research studies.

## Results

### National Policies in advancing CME within medical consortia

The search retrieved 14 policies, of which seven were included in this review. Since 2015, China has made significant strides in developing medical consortia. Initially, hospitals were encouraged to provide consultations and training to PHC institutions, particularly in rural areas. Rural doctors and specialist physicians were offered opportunities to transition to general practice through training programs at medical consortia training bases (30). From 2015 to 2018, national policies gradually established the framework for medical consortia (See Table 2). These policies mandated that tertiary hospitals dispatch experts, conduct joint ward rounds, and participate in case discussions to train GPs, with a focus on diagnosing and treating common and chronic diseases (18, 31–33).

**Table 2:** Timeline of Key national policies on the development of CME in medical consortia.

Time	Title	Main content
2015	Guidance on Promoting the Construction of tiered diagnosis and treatment models (31)	Cultivating GPs through various channels, such as transfer training, standardized general practitioner training, and higher education programs. Promoting remote consultation services, including telemedicine and online education.
2016	Guidelines on the Pilot Work of Establishing Medical Consortia (32)	Encouraging hospitals to guide community health service centers through department construction, clinical teaching, business guidance, teaching rounds, and scientific research.
2017	Guidance on Promoting the Construction and Development of Medical Consortia (18)	Encouraging tertiary hospitals to dispatch management teams and expert teams to PHC institutions. Setting management plans.
2018	Work Program for the Comprehensive Performance Assessment of Medical Consortia (33)	Establishing training evaluation indicators.
2020	Notice on Releasing Management Measures for Medical Consortia (Trial) (19)	Encouraging inter-unit training and research cooperation. Promoting remote medical services, remote consultation, remote rounds, and online education.
2023	Notice on the Pilot Construction of Closely Integrated City Medical Consortia (36)	Physicians from hospitals form medical teams with GPs from in PHC to provide health services.
2025	Notice on the Guidelines for the Informationization of Closely Integrated County Medical Consortia (37)	Establishing the remote CME information system for county medical consortia.

CME, Continuing Medical Education; GPs, General Practitioners; PHC, Primary Health Care.

In 2018, performance evaluation mechanisms were introduced (33). CME completion and improvements in service capabilities were incorporated as key evaluation criteria. Moreover, the use of online platforms to deliver CME courses was encouraged to resolve the conflict between clinical duties and training. From 2019 to 2024, medical consortia facilitated cross-institutional and cross-regional CME collaborations, relaxed CME credit requirements, and alleviated the training burdens on GPs (34, 35). Between 2022 and 2023, policies further promoted collaboration between GPs and specialists within medical teams (36). By 2025, the government began developing the standardized information systems for county-level medical consortia (37). The systems were designed to support remote consultations, enable the sharing of CME resources, and track training outcomes (37).

### Innovations in CME within medical consortia

#### The synergy effect of medical consortia and CME

Mutual benefits were the primary driving force behind sustained CME training within medical consortia. CME was not merely an educational supplement but a critical element of healthcare improvement (38). PHC facilities benefited from specialist support and resource-sharing mechanisms, such as CME, imaging, and pathology services (38–40). Trained GPs were better equipped to make accurate referrals and conduct post-discharge follow-ups (41). The improved reputation of PHC fostered greater patient trust and led to increased local healthcare utilization (42). Tertiary hospitals reduced overcrowding by referring recovered patients back to PHC, thus reserving medical resources for complex cases. Training and collaboration within medical consortia significantly reduced professional burnout among rural GPs, enhancing clinical competence and well-being (43). A well-functioning medical consortia operated effectively without extensive government subsidies (44). Each stakeholder had an inherent incentive to uphold and grow the collaboration (44). A cohort study found that 71.3% of post-training GPs saw 10–30% more outpatients, 57.5% had 10–30% more patients who signed family-doctor contracts, and 83.8% reported higher levels of patient satisfaction (42).

**Figure 3:**
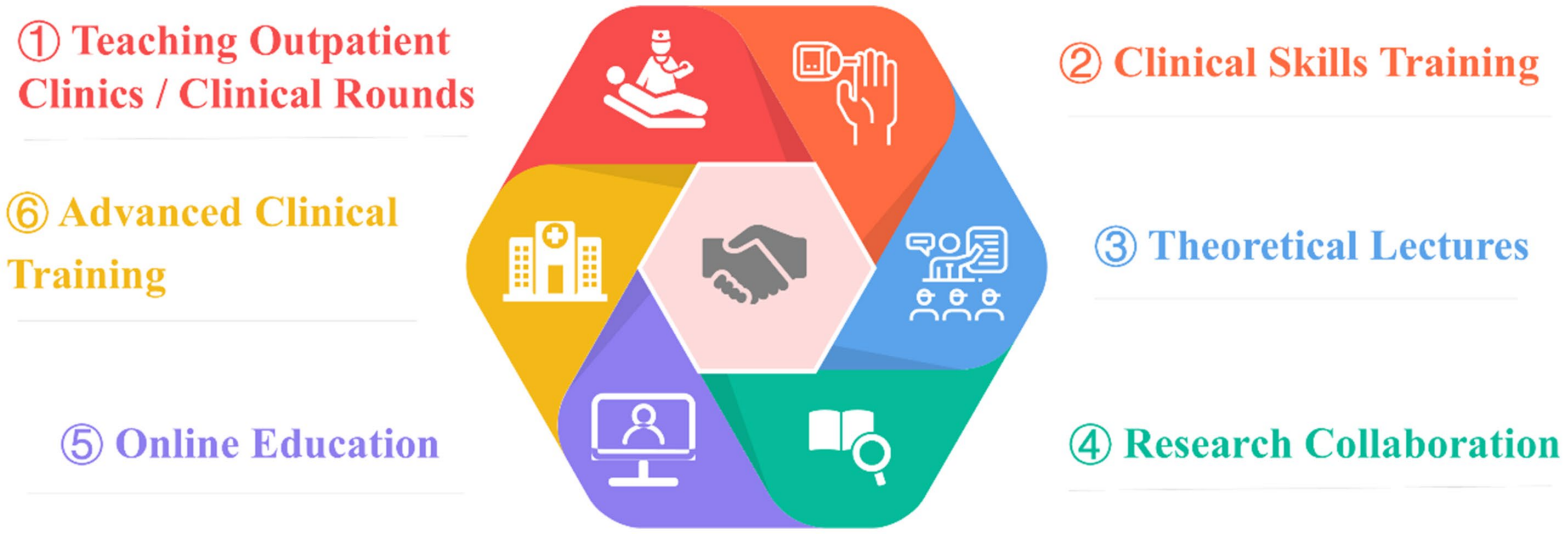
Six key components of CME within medical consortia.

Six components formed the core of the CME model in medical consortia (see Figure 3): (1) Teaching outpatient clinics / Clinical rounds, (2) Clinical skills training, (3) Theoretical lectures, (4) Research collaboration, (5) Online education, and (6) Advanced clinical training. The CME model in medical consortia integrated diverse teaching methods (Table 2), combining theory with practice to address the limitations of traditional approaches (42, 45, 46). It overcame geographical barriers and better met the needs of healthcare professionals across regions and specialties (25, 47).

### Clinical scenario-oriented training

The CME model within medical consortia emphasized training in realistic clinical settings. PHC centers were the main training hubs, with specialists from tertiary hospitals visiting regularly to conduct outpatient clinics, clinical rounds, and on-site teaching (25, 48). This arrangement allowed GPs to learn in real-time, enhancing both their practical skills and clinical decision-making (49). It also fostered specialist involvement in local patient care, thereby reducing unnecessary referrals (39).

### Telemedicine and remote consultation

Comprehensive online education platforms offering a variety of learning modules, interactive sessions, and real-time consultations have improved the accessibility of CME (23, 50). Existing evidence indicated that online education reduced costs, improved access to education, and provided greater flexibility for learners with work and family commitments (28, 51). Many consortia integrated telemedicine platforms to facilitate real-time specialist consultations (25, 37, 42, 52). This approach enabled GPs to receive immediate guidance on diagnosis, treatment options, and patient management, especially in underserved regions (37). GPs benefited from a continuous learning pathway tailored to their specific professional needs, which in turn promoted ongoing professional development (25, 37).

### Flexible training modalities

CME integrated both online and in-person learning (see Table 3). Online platforms broadened access to CME resources, particularly for GPs practicing in rural or remote areas (45, 53). Research collaboration and advanced clinical training further enriched the learning experience (42, 54). This blended-learning model provided flexibility, accommodating varying levels of prior knowledge and expertise (40).

**Table 3:** Strengths and limitations of CME innovations in medical consortia.

Method	Relevant studies	Strengths	Limitations
Clinical scenario-oriented training	An et al., 2020 (25) Zeng et al., 2023 (48) Cai et al., 2023 (66) Gao et al., 2024 (81) Gong et al., 2024 (39)	Facilitates real-time learning in clinical settings, improving practical skills and decision-making, especially for less experienced GPs	Dependent on the availability of specialists and local resources; Complex course design
Telemedicine and remote consultation	An et al., 2020 (25) Ding et al., 2021 (82) Liang et al., 2023 (42) Wang et al., 2024 (53)	Increases access to immediate specialist guidance, particularly in remote areas	Requires stable tech infrastructure; Lack of on-site skills training
Flexible training modalities	Chen et al., 2020 (52) Liao et al., 2020 (45) Sun et al., 2022 (72) He et al., 2022 (40) Liang et al., 2023 (42) Han et al., 2025 (54)	Provides flexible learning options, catering to diverse professional needs	Complex training design; Time-consuming; Suitable for GPs with clinical experience

GPs, General Practitioners.

### Training content in medical consortia

CME programs tailored to the specific needs of GPs not only enhanced retention but also improved patient clinical outcomes (51). CME content focused on managing complex and chronic conditions through collaboration within medical consortia. It enhanced GPs’ clinical knowledge and adherence to evidence-based guidelines, improving patient referrals and follow-up care (28, 55). Research indicated that GPs often sought training that directly enhanced their clinical skills rather than administrative or policy-related content (56–58). Key chronic conditions covered include hypertension, dyslipidemia, type 2 diabetes, coronary heart disease, stroke, chronic obstructive pulmonary disease, osteoporosis, rehabilitation, and periodontal disease (56, 59). Training in clinical skills prioritized essential examinations, common care techniques, doctor-patient communication, shared decision-making, emergency care, cardiopulmonary resuscitation, and wound management (50, 60). New technologies, including wearable devices and artificial intelligence, were increasingly incorporated into the curriculum, improving diagnostic accuracy and healthcare delivery (61).

The public health component addressed vaccination programs, health screening strategies, infectious disease prevention, and maternal and child healthcare (62, 63). Health screenings aimed to identify and refer patients with conditions such as congenital heart disease in children, growth and developmental disorders, hypertension, diabetes, COPD, and common cancers (64, 65). Collaborative frameworks supported prompt responses to infectious disease outbreaks.

### Institutional example: West China hospital-Fangcao community health service center medical consortium

The innovation of West China Hospital—Fangcao Community Health Service Center Medical Consortium (Huaxi-Fangcao medical consortium) was evident in three key areas: organization (staffing), administrative system, mechanism implementation, and four aspects of sharing: personnel, technology, equipment, and information (47). Fangcao Community Health Service Center (Fangcao) provided the necessary infrastructure, medical equipment, and financial support, while West China Hospital offered new medical technology, continuous CME, extensive teaching experience, and management expertise (47). Specialist physicians in endocrinology, respiratory medicine, oncology, dermatology, cardiology, neurology, hematology, nutrition, hepatology, rehabilitation, and mental health conducted daily consultations, teaching clinics, ward rounds, skills training, and research at Fangcao. Trained GPs were able to accurately refer complex cases either to specialist outpatient clinics or directly to West China Hospital for advanced care. Following clinical stabilization, patients were transferred back to Fangcao for ongoing management. Outpatient visits increased from approximately 102,000 in 2021 to approximately 138,000 in 2023, and the number of bidirectional referrals rose from 394 in 2021 to 2,300 in 2023.

Beyond the on-site collaborations, the Huaxi-Fangcao medical consortium jointly organized monthly CME sessions that combined online and face-to-face learning. By April 2025, 48 such training sessions had been conducted, incorporating theoretical lectures, practical skill workshops, and case discussions. GPs and other consortium members were also invited to attend West China Hospital’s regular academic activities, including seminars on frontier research and conferences on complex cases. In parallel, West China Cloud Classroom App was a mobile education platform, providing live broadcasts and recorded lectures to accommodate varied learning needs (25). GPs could additionally pursue advanced clinical training at West China Hospital to further develop their expertise.

### Challenges in implementing CME within medical consortia

The insufficient support and lack of internal training incentives within medical consortia further hindered GPs’ effective participation in CME (66, 67). Training programs largely depended on external incentives, such as CME credits (34, 35). Although some institutions had implemented clinical skill competitions, regular competency assessments, and reward systems to encourage participation, these initiatives proved inadequate (48, 53, 66). Additionally, many GPs lacked clear career advancement pathways and financial incentives, limiting intrinsic motivation to engage in CME (68, 69). The increased workload required to acquire new skills, without corresponding financial remuneration, exacerbated GPs’ reluctance to participate in training (68).

There was a significant shortage of experienced and skilled GP trainers in medical consortia (30). Trainers in China only needed to complete a five-day theoretical course and a course design assignment to be certified (30, 51). Most GP trainers were hospital specialists who lacked experience in managing common medical conditions within the community. This issue was compounded by a limited understanding of the people-centered integrated care (PCIC) concept, which resulted in teaching content that was overly specialized and misaligned with the practical needs of GPs (70, 71). The teaching methods employed rely heavily on traditional and one-dimensional theoretical lectures with little emphasis on interactive and practical approaches, such as problem-based learning, case-based learning, and scenario simulation (45, 66, 72). Specialist doctors involved in mentoring GPs could lack appropriate incentives or performance recognition, which reduced motivation to invest time and resources in CME activities (71).

Several factors hindered the extension of face-to-face CME programs to township areas. There were notable regional disparities in the distribution of medical consortia, with the eastern regions being more developed (23, 73). In rural and remote areas, hospitals capable of providing CME resources were typically unavailable (8, 23). GPs faced constraints such as inadequate transport, limited funding, and a lack of awareness about the need for CME (73, 74). While online CME education has made rapid development in China (37), the practical application of telemedicine infrastructure may be insufficient (75). A survey of 84 PHC institutions across 23 counties in Sichuan Province found that although information hardware facilities were installed, only 47 institutions (56.0%) were connected to other units. Additionally, 34 institutions (41.0%) lacked information technology staff (75).

### Recommendations

The positive performance feedback fosters a sense of accomplishment among GPs, including improvements in patient satisfaction and changes in income (43). From an organizational behavior perspective, existing studies in China showed that intrinsic motivators derived from job characteristics (such as professional growth, training opportunities, and task significance) exerted stronger influence on work outcomes than external incentives like administrative requirements or financial rewards (76). Enhancing CME quality was considered more effective in promoting active participation than relying on mandatory credit systems (76). When GPs perceive work as meaningful, it enhances intrinsic motivation to participate in CME (43). (1) Structured Incentive Mechanisms: CME teaching awards or opportunities of promotions should be conferred upon specialists or GPs who demonstrate excellence in training. (2) Intrinsic motivation reinforcement: Encouraging specialists to engage in multiple-site clinical practice within medical consortia enhances income sources and expands career development opportunities (77). Providing feedback on patient satisfaction and health improvements could help GPs perceive the direct impact of CME participation on their clinical work. (3) Expert-driven training: Inviting renowned experts with medical consortia to deliver training on emerging technologies can increase the credibility and appeal of CME (48, 78).

Training needs assessment is a crucial component for the development of training programs (30, 56). The training needs and skill requirements of GPs vary across different regions of China (79). Medical consortia should evaluate the teaching capabilities of specialists, as well as the satisfaction and effectiveness of training (30). Training content should be tailored to the practical needs of GPs, avoiding excessive specialization (56). Contemporary educational approaches, such as Problem-Based Learning (PBL), Case-Based Learning (CBL), and scenario-based simulations, foster active learning among GPs (80). Specialists mentoring GPs should be proficient in teaching methodologies and understand the core principles of PHC.

To address the low utilization of information equipment in CME, the following strategies are recommended: (1) Establish the online education platform to provide remote courses, thereby reducing reliance on equipment (25). (2) Facilitate equipment sharing among institutions within medical consortia (45, 66). (3) Prioritize the allocation of equipment to PHC institutions with high usage rates and demand, ensuring optimal utilization (75).

### Strengths and limitations

The narrative review not only summarized the synergy and innovation between medical consortia and CME but also analyzed their advantages, challenges, and prospects. This review had several deficiencies. Firstly, the literature search focused on GPs as the primary trainees in CME programs, excluding pharmacists and nurses. Training needs and educational frameworks for pharmacists and nurses were distinct and varied. Secondly, the Huaxi-Fangcao Medical Consortium served as a practical example and policy application, rather than being based on empirically validated research findings. There was little published literature on CME with medical consortia. It was an important and understudied area of medical education. Future implementation science research is needed to advance CME strategies in medical consortia.

## Conclusion

Medical consortia integrate resources and develop innovative CME models, such as scenario-based education, telemedicine, and flexible learning platforms. These models create synergy with medical consortia. However, several challenges remain, including insufficient incentives, a shortage of skilled trainers, and regional disparities. To address these challenges, it is important to strengthen GPs’ intrinsic motivation, promote career development, and adapt strategies to local contexts.
